# Community knowledge, attitude and practices regarding zoonotic viral haemorrhagic fevers in five geo-ecological zones in Tanzania

**DOI:** 10.1186/s12913-023-09317-7

**Published:** 2023-04-12

**Authors:** Sima Rugarabamu, Calvin Sindato, Susan F. Rumisha, Gaspary O. Mwanyika, Gerald Misinzo, Hee Young Lim, Leonard E. G. Mboera

**Affiliations:** 1 SACIDS Foundation for One Health, Sokoine University of Agriculture, Morogoro, Tanzania; 2 Department of Veterinary Microbiology, Parasitology & Biotechnology, Sokoine University of Agriculture, Morogoro, Tanzania; 3 Department of Microbiology & Immunology, Muhimbili University of Health and Allied Sciences, Dar Es Salaam, Tanzania; 4 Tabora Research Centre, National Institute for Medical Research, Tabora, Tanzania; 5 National Institute for Medical Research, Headquarters, Dar Es Salaam, Tanzania; 6 Malaria Atlas Project, Geospatial Health and Development, Telethon Kids Institute, Perth, WA Australia; 7 Mbeya University of Science and Technology, Mbeya, Tanzania; 8 Korea Disease Control and Prevention Agency, National Institute of Health, Osong, Chungchungbukdo Republic of Korea

**Keywords:** Knowledge, Practice, Exposure, Transmission, Viral haemorrhagic fever, Tanzania

## Abstract

**Background:**

Viral haemorrhagic fevers (VHF) cause significant economic and public health impact in Sub-Saharan Africa. Community knowledge, awareness and practices regarding such outbreaks play a pivotal role in their management and prevention. This study was carried out to assess community knowledge, attitude and practices regarding VHF in five geo-ecological zones in Tanzania.

**Methods:**

A cross-sectional study was conducted in Buhigwe, Kalambo, Kyela, Kinondoni, Kilindi, Mvomero, Kondoa and Ukerewe districts representing five geo-ecological zones in Tanzania. Study participants were selected by multistage cluster sampling design. A semi-structured questionnaire was used to collect socio-demographic and information related to knowledge, attitude and practices regarding VHFs. Descriptive statistics and logistic regression were used for the analysis.

**Results:**

A total of 2,965 individuals were involved in the study. Their mean age was 35 (SD ± 18.9) years. Females accounted for 58.2% while males 41.8%. Most of the respondents (70.6%; *n* = 2093) had never heard of VHF, and those who heard, over three quarters (79%) mentioned the radio as their primary source of information. Slightly over a quarter (29.4%) of the respondents were knowledgeable, 25% had a positive attitude, and 17.9% had unfavourable practice habits. The level of knowledge varied between occupation and education levels (*P* < 0.005). Most participants were likely to interact with a VHF survivor or take care of a person suffering from VHF (75%) or visit areas with known VHF (73%). There were increased odds of having poor practice among participants aged 36–45 years (AOR: 3.566, 95% CI: 1.593–7.821) and those living in Western, North-Eastern and Lake Victoria zones (AOR: 2.529, 95% CI: 1.071–6.657; AOR: 2.639, 95% CI: 1.130–7.580 AOR: 2.248, 95% CI: 1.073–3.844, respectively).

**Conclusion:**

Overall, the knowledge on VHF among communities is low, while a large proportion of individuals in the community are involved in activities that expose them to the disease pathogens in Tanzania. These findings highlight the need for strengthening health educational and promotion efforts on VHF targeting specific populations.

**Supplementary Information:**

The online version contains supplementary material available at 10.1186/s12913-023-09317-7.

## Introduction

Viral haemorrhagic fevers (VHFs) are severe viral infections characterised by multi-organ failure and haemorrhages resulting in high morbidity and mortality rates [1] (Pigott, 2005). They cause severe epidemics resulting in catastrophic situations that can interrupt everyday life, livelihoods or social structure [2, 3]. VHFs of public health importance in Sub-Saharan Africa include, Ebola virus Disease (EVD), Marburg virus disease (MVD), Crimean-Congo haemorrhagic fever (CCHF), Rift Valley fever (RVF), Lassa and yellow fever (YF) [4–7]. Available evidence indicates that RVF and CCHF have been reported in Tanzania [8, 9]. Although no clinical cases of EVD, MVD or YF have been reported in the country, a recent study has reported the presence of immunoglobulin M (IgM) and IgG antibodies against the viruses across the country [10].

Viruses causing haemorrhagic fever are transmitted to humans when the activities of the human host and infected reservoir hosts or vectors overlap. The exploitation of new ecological niches by human beings and increased travel and trade as well as climate change have promoted the emergence or re-emergence of VHF around the world [11, 12]. Direct man-made environmental change may also impact ecology and increase interactions between vectors, wild animals, and humans [13]. The consumption of wild animals (or bushmeat) and animal products such as raw milk and blood is an important driver of VHF emergence [14]. Wild animals are indeed important reservoirs of VHF, and most of the VHF pathogens originate from wildlife [15]. Animal farming plays a significant role in the emergence and the spread of VHFs such as RVF and CCHF [2]. Mining, hunting, and intensive farming involve significant risks in exposure to VHF reservoirs through means other than commercial production [16].

VHF outbreaks have been reported in communities with limited knowledge and increased exposure practices [17, 18]. Knowledge and practices can be improved by providing appropriate information on disease recognition, transmission, prevention and management [19]. Disease prevention education and awareness strategy through traditional and social media have significantly improved knowledge and practice regarding Lassa fever in Nigeria [20]. However, there is a marked gap between VHF awareness and exposure practice in some Sub-Saharan Africa countries. Studies have shown that although most people in Sudan, Democratic Republic of the Congo and Uganda were described to be aware of EVD and RVF, their preventative practice levels were poor [21, 22].

The frequent outbreaks of viral haemorrhagic fevers in Sub-Saharan Africa highlight the importance of developing national strategy for prevention and management of outbreaks. These include the need for strong health education and promotion programmes. To date, few studies have evaluated knowledge, attitudes, and perceptions of VHF in Tanzania. Understanding the community knowledge, attitudes, and practices is important in outbreak prevention and control. In recognition of the growing threats of VHFs in the country, this study was carried out to assess the community knowledge, attitudes, and exposure practices on zoonotic VHFs in diverse five ecological zones in Tanzania.

## Methods

### Study areas design and population

This community-based cross-sectional study was carried out from April to November 2018. Respondents were selected using a multistage cluster sampling design. The country was divided into five ecological zones based on rainfall pattern, vegetation, land use pattern and altitude. Furthermore, these areas have previously been described as suitable habitat for primary vectors, which can contribute to the occurrence of mosquito- and tick-borne viral diseases [23]. Zone 1 comprised of the western parts of the country with tropical forest, unimodal rainfall pattern, and altitude < 2,300 m above sea level (a.s.l). In this zone, Buhigwe (in Kigoma Region) and Kalambo (in Rukwa Region) were selected as study districts. Zone 2 included the Southern highlands regions characterised by high precipitations, bimodal rainfall pattern, tropical forest and elevation > 2,300 m a.s.l. In this zone, Kyela district (in Mbeya Region) was selected. Zone 3 comprised of the north-eastern regions, with bimodal rainfall pattern and an elevation of < 1,800 m a.s.l. In this zone, Kinondoni (in Dar es Salaam Region) and Kilindi (in Tanga Region) districts were selected. Zone 4 covered the central regions of the country, characterised by moderate precipitation and unimodal rainfall pattern. In this zone, the selected districts were Mvomero (Morogoro Region) and Kondoa (Dodoma Region). Zone 5 comprised of the Lake Victoria zone, characterised by bimodal rainfall pattern; and Ukerewe (Mwanza Region) was the selected study district. In each district, three wards and nine villages were selected to account for local ecological biodiversity variations.

Based on the variations of the ecology in each zone, the number of individuals to be included in the study was calculated independently for each zone. Then population-weighted samples were used to split among the districts. The sample size for this study was calculated based on the conservative prevalence of peoples’ knowledge about VHF of 50%, a desired absolute precision of 5%, and a confidence level of 95%. The sample size was adjusted for clustering between districts by a design effect factor of 1.5. A contingency of 10% was considered to account for nonresponses, refusal or missingness. The minimum estimated sample size was 2,840 individuals. Within each district, individuals were evenly distributed between sampled wards and villages. Individuals over the age of 18 who voluntarily consented were interviewed.

### Data collection

A pre-tested semi-structured questionnaire installed in smartphones with digital data collection tool was used to collect sociodemographic and other relevant data [24]. The questionnaire consisted of four sections that addressed the (i) socio-demographic information; (ii) participants’ knowledge of the VHFs, source of information; (iii) attitudes towards VHF prevention; and (iv) exposure and risk practices related to VHFs. Trained research assistants administered the questionnaires in Kiswahili, the national language in Tanzania. To avoid stigma and improve participation, all interviews were organised privately.

### Data management and analysis

Each response was scored 1 for the correct answer while 0 for the wrong or “don’t know” response. Knowledge about VHF was graded on 0 to 3, with 0 being the lowest and 3 being the highest. Participants’ scoring from 2 or 3 was considered “good knowledge” while 0 or 1 indicated “poor knowledge”. Attitude about VHF score was reversed graded on a scale of 0 to 3, with a score of 0 and 1 indicating “favourable attitude” and a score of 2 or 3 indicating “unfavourable attitude”. Finally, risk practice against VHF was measured on a scale of 0 to 22. Participants who received a score of 11 or higher were deemed to have optimal/good practice, whereas below 11 were considered to have at-risk/poor practice (Supplement 1).

Data analysis was performed using the Statistical Package for the Social Sciences (SPSS) version 27.0. Descriptive analysis was performed, and the results were reported as frequencies and percentages. Bivariable and multivariable logistic regression analyses were conducted to determine the relationship between socio-demographic characteristics and knowledge, attitude and practice levels. All variables with a *p*-value < 0.2 from the bivariable analysis were entered into the multivariable model. Possible associations were measured using an adjusted odds ratio (AOR) with 95% CI, and the *p*-value of less than 0.05 was considered statistically significant.

## Results

### Socio-demographic characteristics of the respondents

A total of 2,965 participants were interviewed from 24 wards and 48 villages of eight districts. The mean age was 35 (SD ± 18.9) years. Over half 1,726 (58.2%) of the study participants were females, and most respondents 1,826 (61.6%) had primary school education. The majority of the respondents were farmers. Half 1,803 (60.8%) were farmers (Table 1).

**Table 1:** Sociodemographic characteristics of the respondents by district

Variable	Response	Zone 1		Zone 2	Zone 3		Zone 4		Zone 5	Total	*P*-value
		**Buhigwe**	**Kalambo**	**Kyela**	**Kinondoni**	**Kilindi**	**Kondoa**	**Mvomero**	Ukerewe		
		n (%)	n (%)	n (%)	n (%)	n (%)	n (%)	n (%)	n (%)	n (%)	
**Sex**	Female	207 (56.2)	209 (59.2)	207 (55.2)	221 (60.2)	203 (54.4)	223 (59%)	225 (64%)	231 (65%)	1,726 (58.2%)	
	Male	161 (43.8)	164 (40.8)	168 (44.8)	146 (39.8)	170 (45.6)	148 (41%)	142 (36%)	140 (35%)	1,239 (41.8%)	0.00
**Age**	18–35	132 (33.6)	142 (39.6)	142 (37.8)	134 (36.5)	141 (37.8)	135 (36.3)	135 (36.7)	135 (36.4)	1,096 (37.0%)	
	36–45	126 (34.2)	123 (33.1)	125 (33.4)	125 (34.0)	124 (33.2)	126 (34.0)	122 (33.2)	126 (33.9)	997 (33.6%)	
	46- 65	110 (32.2)	108 (29.1)	108 (28.2)	108 (29.5)	108 (29)	110 (29.7)	110 (30.1)	110 (29.7)	872 (29.4%)	0.00
**Education**	None	128 (34.7)	107 (28.8)	84 (22.4)	42 (11.5)	131 (35.1)	76 (20.4)	87 (23.7)	80 (21.5)	759 (25.6%)	
	Primary	225 (61.1)	231 (62.7)	213 (56.7)	226 (61.5)	208 (55.8)	242 (65.3)	236 (64.4)	256 (69.2)	1,826 (61.6%)	
	≥ Secondary	20 (5.6)	32 (8.5)	85 (25.2)	103 (30.7)	49 (12.9)	53 (14.3)	56 (15.3)	47 (12.4)	458 (15.4%)	0.00
**Occupation**	Farmer	251 (68.1)	278 (74.6)	254 (67.7)	277 (21.1)	59 (75.0)	228 (61.2)	201 (57.6)	240 (64.6)	1,803 (60.8%)	
	Employed	15 (4.2)	26 (6.8)	40 (10.7)	25 (52.0)	72 (19.5)	38 (10.1)	38 (10.2)	57 (15.4)	461 (15.6%)	0.02
	Student	25 (6.9)	6 (1.7)	29 (7.7)	35 (9.6)	19 (5.2)	45 (12.2)	31 (8.5}	40 (10.8)	228 (7.7%)	
	Others	77 (20.8)	63 (16.9)	52 (13.9)	65 (17.3)	38 (10.3)	60 (16.3)	87 (23.7)	34 (9.2)	473 (15.9%)	

None = did not obtain formal education

### Knowledge about VHF

Table 2 shows the responses of participants on knowledge on VHF. Of the participants, 872 (29.4%) were familiar with at least one VHF and reported VHFs were EVD. RVF, CCHF, YF were least mentioned VHF diseases. Other diseases mentioned were dengue 87 (10%), chikungunya 43 (5%) and malaria 19(2.2%). According to 688 (79%) respondents, radio was the main source of information about VHFs. The majority of the respondents were of the (*n* = 714, 81.8%) opinion that VHF is preventable (Table 2).

**Table 2:** Knowledge of study participants about VHF

Variable	Category	Frequency	Percentage (%)
Heard of VHF (*N* = 2965)	Yes	872	29.4
	No	2093	70.6
VHF disease heard (*N* = 872)**	Ebola	460	52.6
	Rift Valley Fever	112	12.8
	Yellow Fever	44	5.0
	Crimean-Congo Haemorrhagic Fever	88	10.1
	Others	150	17.2
	Don’t know	23	2.6
Source of Information (*N* = 872)**	Television	213	24.4
	Radio	688	79.0
	Family	45	5.1
	Friend	22	2.5
	School	180	20.6
	Newspaper	75	8.6
Know how VHF can be prevented (*N* = 872) **	Yes	714	81.9
	No	158	18.1

Multiple responses were allowed

** Number of participants who had only heard of VHF

### Factors associated with knowledge

Bivariable analysis revealed that education and occupation were highly associated with the knowledge index. The multivariable regression analysis indicated that occupation and education were the two independent predictors of the knowledge index. The employed participants (AOR: 5.82, 95% CI: 1.27–6.48) were more likely to have better knowledge than other participants. Respondents with highest education level had a great understanding of the VHFs compared to their counterparts (AOR: 2.03, 95% CI: 1.27–6.48). Respondents’ knowledge did not statistically vary between zones (Table 3).

**Table 3:** Association between knowledge level and socio-demographic characteristics

**Variable**		**Bivariate Analysis**		**Multivariate Analysis**	
		**Odds ratio (95% CI)**	***P*-value**	**Adjusted odds ratio (95% CI)**	***P*-value**
Sex	Female	1			
	Male	1.097 (0.438–2.745)	0.734		
Age (years)	18–35	1			
	36–45	1.061 (0.410–2.743)	0.903		
	46- 65	1.026 (0.361–2.918)	0.952		
Education	None	1		1	
	Primary	1.02(0.63–1.42)	0.229	1.02(0.71–1.36)	0.243
	≥ Secondary	2.06(1.53–2.86)	0.024*	2.03(1.39–2.98)	0.0023*
Occupation	Farmer	3.000(0.968–6.302)	0.027*	1.818 (0.357–9.255)	0.375
	Employed	4.286(1.223–5.022)	0.022*	5.82(1.27–6.48)	0.021*
	Student	0.079(0.009–0.672)	0.020*	0.090(0.008–1.023)	0.054
	Others	1		1	
Zones	Western	1.061 (0.410–2.743)	0.229		
	Southern Highlands	2.842 (0.326–3.792)	0.228		
	North-Eastern	1.026 (0.361–2.918)	0.264		
	Central	1.928 (0.493–3.485)	0.962		
	Lake Victoria	1.913 (0.827–4.422)	0.224		

^*^P-value < *0.05*

### Attitude towards VHF

The disease fatality was unknown to almost over half of the study participants. Three quarter of the respondents (= n 654, 75%) said they would either interact or take care of a person suffering from VHF. A large proportion of respondents (*n* = 637, 73%) said that they would visit regions known to experience VHF outbreaks (Table 4).

**Table 4:** Respondents’ Attitudes toward VHF (*n* = 872)

Variable	Response	Frequency	Percentage
Living with a person who suffered VHF	Yes	654	75.0
	No	201	23.1
	I don’t know	17	1.9
Taking care of a person with VHF	Yes	654	75.0
	No	183	21.0
	Don’t Know	35	4.0
Travel to regions with VHF	Yes	637	73.0
	No	192	22.0
	Don’t know	44	5.0

### Factors associated with attitude

Age and sex were the two independent predictor variables of attitude towards VHF. Male participants (AOR: 2.72, 95% CI: 1.07–6.89) were more likely to have an unfavourable attitude than females. Participants aged 36 years and above had significantly less favourable attitude than their peers (AOR: 2.95, 95% CI: 1.25–8.27). There was no association between either education level, occupation or zone and attitude (Table 5).

**Table 5:** Association between attitude and socio-demographic characteristics

**Variable**		**Bivariate Analysis**		**Multivariate Analysis**	
		**Odds ratio (95% CI)**	***p*-value**	**Adjusted odds ratio (95% CI)**	***p*-value**
Sex	Female	1		1	
	Male	2.168 (1.98–5.05)	0.050*	2.72 (1.07–6.89)	0.048*
Age (years)	18–35	1		1	
	36–45	2.600 (1.988–6.769)	0.040*	2.95 (1.25–8.27)	0.038*
	46- 65	1		1	
Education	None	1			
	Primary	1.376 (0.438–4.020)	0.078		
	≥ Secondary	0.951 (0.457–2.466)	0.945		
Occupation	Farmer	1.832 (0.805–4.167)	0.149		
	Employed	1	1		
	Student	0.976 (0.356–2.408)	0.770		
	Others	0.876 (0.211–2.312)	0.650		
Zones	Western	1.034 (0.309–2.651)	0.242		
	Southern Highlands	2.542 (0.126–7.692)	0.326		
	North-eastern	1.032 (0.341–2.618)	0.264		
	Central	1.528 (0.493–4.465)	0.663		
	Lake Victoria	1.712 (0.727–3.422)	0.424		

### VHF exposure and risk practices

**Fig. 1:**
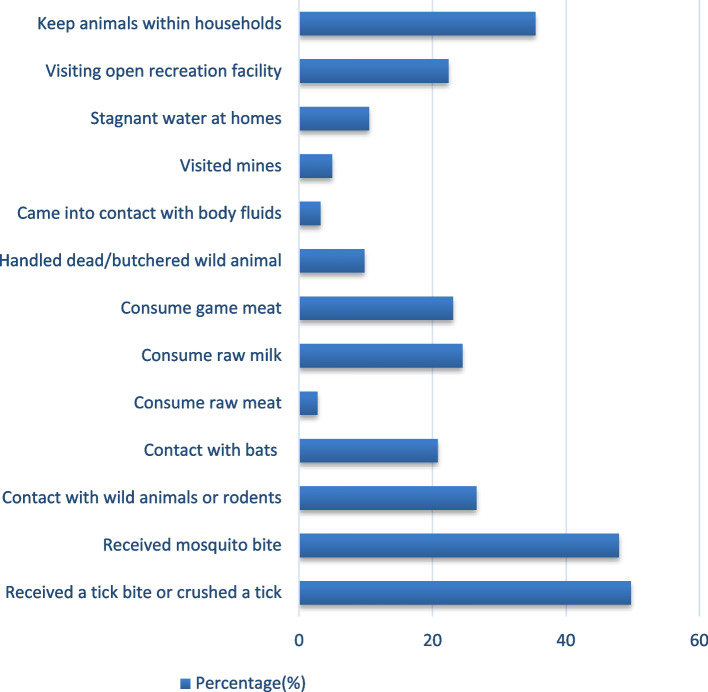
Percentage distribution of exposure practise scores

About half of participants (*n* = 1,473, 49.7%) admitted to have had received a tick bite, removed a tick and/or crushed a tick with their bare hands during a period of six month before the study. About half of the respondents (1*n* = 420, 47.9%) reported having received a mosquito bite during the previous week. The majority of the participants (*n* = 1,897, 64%) were using mosquito nets to protect themselves from mosquito bites. Other exposure practices identified included keeping animals within the household 1,050 (35.4%), contact with wild animals or rodents 788 (26.6%), consumption of raw milk 726 (24.5%), frequent visits of outdoor recreation facilities 664 (22.4%) and contact with bats 471 (15.9%) (Fig. 1). Overall, 22% of the participants scored below the mean. The mean exposure practice score was 9.70 (SD = 1.17) out of a maximum of 22 points, indicating a low level of exposure practice.

### Participants’ practices toward VHF exposure

Sex, age and residence of the respondents were significantly associated with the high-risk practices. There were increased odds of VHF exposure and risk practices among males (AOR: 2.95, 95% CI: 1.25– 6.27), participants aged 36–45 years (AOR: 3.58, 95% CI: 1.59– 7.82) and those who reside in Western, North-Eastern and Lake Victoria zone (AOR: 2.53, 95% CI: 1.07–6.66; AOR: 2.64, 95% CI: 1.13–7.58 AOR: 2.25, 95% CI: 1.07–3.84) than their peers in the other zones (Table 6).

**Table 6:** Association between practise and socio-demographic characteristics

**Variable**		**Bivariate Analysis**		**Multivariate Analysis**	
		**Odds ratio (95% CI)**	***P*-value**	**Adjusted odds ratio (95% CI)**	***P*-value**
Sex	Female	4.08 (0.47– 5.43)	0.202	4.82 (0.38–6.59)	0.228
	Male	2.95 (1.27–5.27)	0.005*	2.95 (1.25–6.27)	0.046*
Age (years)	18–35	1.04 (0.36–3.06)	0.938	1.34 (0.13–1.45)	0.300
	36–45	4.08 (1.47– 5.43)	0.040*	3.58 (1.59–7.82)	0.006*
	46- 65	2.95 (1.26–5.27)	0.005*	2.89 (0.09–6.023)	0.052
Education	None	1			
	Primary	1.376 (0.438–4.020)	0.078		
	≥ Secondary	1.133 (0.262–3.99)	0.063		
Occupation	Farmer	1.832 (0.805–4.167)	0.449		
	Employed	1	1		
	Student	0.98 (0.36–2.41)	0.770		
	Others	0.88 (0.21–2.31)	0.650		
Zone	Western	2.44 (1.05–5.66)	0.038 *	2.53 (1.17–6.66)	0.039*
	Southern Highlands	2.35 (0.23–4.49)	0.465	2.07 (0.44–2.85)	0.865
	North-eastern	2.74 (1.14–6.58)	0.024 *	2.64 (1.13–7.58)	0.022*
	Central	1.01 (0.22 –2.32)	0.3440	1.00 (0.09–4.67)	0.388
	Lake Victoria	2.32 (1.11–3.94)	0.035*	2.25 (1.97–3.84)	0.024*

^*^P-value < 0.05

## Discussion

This study aimed to determine the level of community knowledge and practices in Tanzania following the increased spread of VHFs in Sub-Saharan countries. Only about a quarter of the respondents had heard of VHFs. About three quarters of the respondents were not aware of VHFs, indicating that majority of the people in Tanzania are not familiar with the diseases. The level of awareness observed in the current study is lower than that reported in previous study among the pastoral community in previous studies in Tanzania and the general population in Uganda, Kenya, and DRC [17, 25–27]. RVF is not uncommon in some pastoral communities in Tanzania, which could explain why this group is more aware of VHF than other communities [17].

The most commonly known VHF among the community in this study was EVD. The knowledge about EVD was likely to be associated with the fact it has occurred in the Democratic Republic of the Congo during this study period, and several national and international communication channels have raised awareness about the disease [28]. In a similar study in West Africa, most members of the community could identify EVD as the most known VHF [18]. These findings suggest that knowledge about EVD is the general consequence of heightened awareness that the recent outbreaks in West Africa and DRC have created around the globe. EVD has sharpened people’s knowledge and practice because it has dominated sub-Saharan Africa’s daily lives for so long [29]. From our study, most of those who knew VHF named mass media, specifically radio, as their main sources of information. Similar findings have been reported in previous studies in Tanzania and elsewhere in Africa [30, 31].This indicates that mass media channels, are important sources of information. Three-quarters of the study participants had unfavourable attitudes toward VHF. This could be attributed to the fact that the majority of them had limited knowledge about the diseases. Our findings contrast with those previously reported from VHF endemic regions [18].

This study found that occupation and education were important determinants of good VHF knowledge. A similar finding was reported from Tanzania, Sierra Leone, Kenya and Uganda [23, 24, 30]. Employed participants were more likely to have good knowledge. Respondents who had education were more knowledgeable of the disease than their peers. This finding is concurrent with a study conducted in Guinea where educated people had better knowledge on EVD than those not educated [18]. In a study in Ghana, people with no education, those not working were reported to be the least knowledgeable in EVD [31]. Educational level, monthly income has been reported to be significantly associated with practices as regards to infectious disease prevention and control [32].

Seventy-three percent (73%) of respondents said they had no fear of traveling to outbreak areas; this attitude could be attributed to the fact that few outbreaks have been reported in the regions, and the majority of respondents know very little about the disease. Some community members, such as health care workers, may hold opposing viewpoints. In an Ethiopian study, 78% of health case workers were afraid of VHF when compared to other community members [33].

Males and adults of 36–45 years old and people living in Western, North-Eastern and Lake Victoria zones were at higher odds of having poor practices with regard to VHFs. The possible reason for this might be because participants in this group are in the adulthood stage, which allows them to be socially proactive and on-demand of exposure practice such as taking care of sick, hunting or mining. The finding also indicates that the areas with odds of exposure practise have characterisation of risk of occurrence of mosquito- and tick-borne viral diseases in Tanzania [23, 34, 35]. Moreover, a recent serological study in Tanzania has shown that the districts in the three zones to have relatively higher prevalence of VHF antibodies [10].

The findings of this study have highlighted other exposure practices that would put the community at risk of VHF. These include tick bites, crushing ticks with bare hands, mosquito bites, contact with wild animals including rodents, birds and bats. Other identified practices were handling and butchering of wild animals, contact with the body fluids, or direct contact with dead animals/individuals. Even though few respondents practised this, it takes only a single person to be exposed to a virus before the disease would spread to the community. As a result, when it comes to VHF transmission and spread, a low percentage of risk practice would appear to be sufficient for an epidemic to occur. Educational programs should encourage avoiding exposure practices that would put a community at risk of disease. In addition, a heightened awareness of VHF may lead to more rapid identification of outbreaks and may result in decreased transmission.

Our study showed that about one-fifth of respondents consumed raw meat, raw milk, raw blood, game (including primate) meat, which are among the risk practices for the occurrence of RVF and EVD [21]. Some studies have reported exposure to raw milk, raw meat or blood as a potential route for RVF transmission [36–38]. Animal products, regardless of status, is an important part of the diet in some communities in Tanzania. Milk and meat are mostly raw, fermented, and rarely boiled [39, 40]. Overall, the findings emphasise the importance of educational campaigns and endless activities towards behavioural change in communities living in risk areas.

## Conclusions

Based on the findings, it can be concluded that there is a low level of community knowledge regarding VHF despite higher exposure risk behaviour in Tanzania. Only about a third of the study participants had previously heard about VHF; and the majority have heard of EVD. Occupation and education were the most important predictors of the knowledge index. Respondents with highest education level had a great understanding of the VHFs than those with low education levels. Male participants were more likely to have an unfavourable attitude than females. Sex, age and residence of the respondents were significantly associated with the high-risk practices. These findings call for heightening VHF awareness and prevention campaigns in Tanzania.

## Supplementary Information

**Additional file 1: Supplement 1.** Structured questionnaire.

## Data Availability

The datasets used and analysed during the current study available from the corresponding author on reasonable request.
