# Supplementary material for: Community knowledge, attitude and practices regarding zoonotic viral haemorrhagic fevers in five geo-ecological zones in Tanzania
Source: BMC Health Serv Res. 2023 Apr 12;23:360. doi: 10.1186/s12913-023-09317-7 (PMC10091607; doi:10.1186/s12913-023-09317-7)
Supplement: Supplementary file 1 — Additional file 1: Supplement 1. Structured questionnaire. [file 12913_2023_9317_MOESM1_ESM.docx]

Structured questionnaire

Community knowledge, attitude and practices regarding zoonotic viral haemorrhagic fevers in five geo-ecological zones in Tanzania

Interviewer name:_________________________________________________

| **A. Identification Information** *(To be filled by Interviewer)* | |
| --- | --- |
| A.1 Region |  |
| A.2 District |  |
| A.3 Village/Street |  |
| A.4 Date of interview/sample collection (dd/mm/yyyy) | [__][__]/[__][__]/[__][__][__][__] |
| A.5 Place of sample/data collection | 1. Household [___] 2. Facility [___] |
| A.6. If A.5 = “2”; mention the name and level of facility | 6.0. Facility name _________________________________  Level: 6.1. Dispensary [___], 6.2. Health Centre [__], 6.3. Hospital [__] |
| **B. Participant details** *(To be filled by Interviewer)* | |
| B.0 Participant ID (Take from names sheet; use it to label the sample). The first three letters represent the district name ***(Refer A.2)*** followed by three numbers representing the ID of participant). | [__][__][__] [__][__][__] |
| B.1 Age (years)/(months) | [__][__][__] [_ ] |
| B.2 Sex | 1. Male [__] 2. Female [__] |
| B.3 Place of Living (Village/street) | \|  \| \| --- \| |
| B.4 How long have you lived in this village/street? | Years [___] Months [____] |
| B.5 Occupation (multiple selection) | 1=Livestock farming  2=Crop farming  3=Fishing  4=Hunting  3=Informal sector (Daily labored/self-employed)  4=Formal employment (Public service, /private sector/NGOs)  6=Student (primary/secondary)  7=Business/trader  8=Mining  8=Others, specify _ __ ____________ |
| B.6 Highest Educational level of the respondent | 1= Primary school; 2=Secondary school  3= College/University; 4=Vocational training school; 5= Adult education; 6=None |
| **C. Knowledge (***to be filled by Interviewer)* | |
| C.1 Have you heard of VHF before (prior to this interview)? | 1=Yes  2= No |
| C.2 VHF disease heard? | 1._____________________________ |
| C.3 Source of Information | 1._____________________________ |
| C.4 Do you know how VHF can be prevented? | 1=Yes  2= No |
| **D. Attitude** | |
| D.1. Can you live with a person who suffered VHF?  1. Strongly disagree **[** __] 2. Disagree [__] 3. Neutral [__] 4.Agree [__] 5. Strongly agree [__]  D.2. Can you care a person with VHF?  1. Strongly disagree [ __] 2. Disagree [__] 3. Neutral [__] 4. Agree [__] 5. Strongly agree [__]  D.3. Can you travel to regions with VHF outbreak?  1. Strongly disagree [ __] 2. Disagree [__] 3. Neutral [__] 4. Agree [__] 5. Strongly agree [__] | |
|  | |
| **E. Exposure Practises risk assessment (***To be filled by researcher***)** | |
| Has participant  E.1 Lived or worked in basic rural conditions where VHF is endemic? [_ ] Yes [_] No [_] NK | |
| E.2 Received a tick bite &/or removed a tick &/or crushed a tick with their bare hands [_ ] Yes [_] No [_] NK | |
| E.3 Travelled to a rural environment where contact with livestock or rodents? [_ ] Yes [_] No [_] NK | |
| E.4 Swept/cleaned dust which could have been contaminated by rats? [_ ] Yes [_] No [_] NK | |
| E.5 Handled or butchered dead primates/been involved in drying, smoking or consuming their meat [_] Yes [_] No [_] NK | |
| E.6 Come into contact with the body fluids of, or had direct contact with, alive or dead individual or animal, known or strongly suspected to have VHF, [_] Yes [_] No [_] NK | |
| E.7 Visited caves or mines? [_] Yes [_] No [_] NK | |
| E.8 Had close contact with alive or dead individual known or strongly suspected to have VHF [_] Yes [_] No [_] NK | |
| E.9 Come into contact with body fluids of a live or dead individual known or strongly suspected to have VHF either directly, e.g. handled blood, urine, or indirectly [_] Yes [_] No [_] NK | |
| E.10 Handled clinical/laboratory specimens (blood, urine, faeces, tissues, laboratory cultures) from a live or dead individual or animal known or strongly suspected to have VHF?  E.11 Presence of piped water at home [ ] Yes [ ] No [ ] NK  E.12 Frequent visit recreation facilities [ ] Yes [ ] No [ ] NK  E.13 Use mosquito net [ ] Yes [ ] No [ ] NK  E.14 Use mosquito repellants [ ] Yes [ ] No [ ] NK | |
